# Southern Surgeons Abroad

**Published:** 1864-04

**Authors:** 


*Southern Surgeons Abroad.*

Doctor Marion Sims is now practising in Paris, and as the improvements which he has introduced into what may be called the surgery of females have revolutionized that department of the art all over the world, and his procedures have formed the basis of a new and happy era in the treatment of the series of fistulæ and other diseases of women which were previously incurable, it will be interesting to give some particulars of what he has been doing here surgically since, like many other Americans, he has sought a home in Europe.

Dr. Sims had already achieved a great reputation in America and England by his introduction of silver wire suture—certainly one of the most important surgical improvements of the century; and by his immediate application of this discovery to the cure of all kinds of fistulæ and clefts in the mucous canals. The success with which he operates on deformities and deficiencies of this kind was not, however, at first fully appreciated at Paris; and when he came over, he was asked to operate on some of the most difficult cases of prolapse of the bladder from sloughing of the tissues, and utero-vesical and vaginal fistulæ, which were known to exist in this capital. He had a triumphant success in these cases, which had been abandoned as hopeless, but which he cured with a single operation; and his reputation soon extended so greatly that he was summoned to the highest and most important personages, and the Emperor has given him a special permission to practice in Paris. It is no breach of confidence, since the fact has already appeared in print, to mention that Dr. Sims has been consulted by the Empress, whose general health is good, but who is known to be delicate. —*Paris Letter—Lancet, March*, 1864.

